# Manipulating the Temperature of Sulfurization to Synthesize α-NiS Nanosphere Film for Long-Term Preservation of Non-enzymatic Glucose Sensors

**DOI:** 10.1186/s11671-018-2511-8

**Published:** 2018-04-19

**Authors:** Hsien-Sheng Lin, Jen-Bin Shi, Cheng-Ming Peng, Bo-Chi Zheng, Fu-Chou Cheng, Ming-Way Lee, Hsuan-Wei Lee, Po-Feng Wu, Yi-Jui Liu

**Affiliations:** 1 Ph.D. Program in Electrical and Communications Engineering, Feng Chia University, 100, Wen-Hwa Rd, Seatwen, Taichung, 40724 Taiwan; 2 Department of Electronic Engineering, Feng Chia University, 100, Wen-Hwa Rd., Seatwen, Taichung, 40724 Taiwan; 3 Da Vinci Minimally Invasive Surgery Center, Chung Shan Medical University Hospital, No.110, Sec.1, Chien-Kuo N. Rd., Taichung, 40201 Taiwan; 4 Department of Medical Research, Taichung Veterans General Hospital, No. 160, 3rd Section, Taichung Harbor Road, Taichung, 40705 Taiwan; 5 Department of Physics, Institute of Nanoscience, National Chung Hsing University, 250 Kuo Kuang Road, Taichung, 40227 Taiwan; 6 College of General Education, No. 1018, Sec. 6, Taiwan Boulevard, Shalu District, Taichung, 43302 Taiwan; 7 Department of Automatic Control Engineering, Feng Chia University, No.100, Wenhwa Rd., Seatwen, Taichung, 40724 Taiwan

**Keywords:** Nanosphere, α-NiS, Electrodeposited, Non-enzymatic, Glucose, Sensor

## Abstract

In this study, alpha nickel sulfide (α-NiS) nanosphere films have been successfully synthesized by electroplating the nickel nanosheet film on the indium tin oxide (ITO) glass substrate and sulfuring nickel-coated ITO glass substrate. First, we electrodeposited the nickel nanosheet films on the ITO glass substrates which were cut into a 0.5 × 1 cm^2^ size. Second, the nanosheet nickel films were annealed in vacuum-sealed glass ampoules with sulfur sheets at different annealing temperatures (300, 400, and 500 °C) for 4 h in vacuum-sealed glass ampoules. The α-NiS films were investigated by using X-ray diffraction (XRD), variable vacuum scanning electron microscopy (VVSEM), field emission scanning electron microscopy/energy dispersive spectrometer (FE-SEM/EDS), cyclic voltammogram (CV), electrochemical impedance spectroscopy (EIS), ultraviolet/visible/near-infrared (UV/Visible/NIR) spectra, and photoluminescence (PL) spectra. Many nanospheres were observed on the surface of the α-NiS films at the annealing temperature 400 °C for 4 h. We also used the high-resolution transmission electron microscopy (HR-TEM) for the analysis of the α-NiS nanospheres. We demonstrated that our α-NiS nanosphere film had a linear current response to different glucose concentrations. Additionally, our α-NiS nanosphere films were preserved at room temperature for five and a half years and were still useful for detecting glucose at low concentration.

## Background

Over the last decade, nickel sulfide (NiS) has been accepted as having good conductivity. It can be melted as a cathode material for lithium rechargeable batteries [1–3]. Furthermore, NiS has been applied to solar storage [4, 5]. It has also been proofed to have excellent properties for application in photocatalyst [6, 7]. NiS film can also be used for non-enzymatic glucose sensor [8, 9]. About glucose detection, many sensing methods for detecting glucose have been developed. The most widely used and historically significant methods included copper iodometry, high-performance liquid chromatography (HPLC), glucose oxidase (GC), capillary zone electrophoresis (CZE), and non-enzymatic glucose sensor [10]. A non-enzymatic glucose sensor will be an important application for glucose detection in the future [11]. We are interested in synthesizing NiS film and research this kind of material for one of the important applications of non-enzymatic glucose sensor. In the sensor preservation study, the non-enzymatic glucose sensor can preserved more time than enzymatic glucose sensor [12]. In this paper, we will describe the synthesis process of α-NiS film and demonstrate our specimens which can be used in detecting glucose by cyclic voltammogram (CV) measurements and amperometry. We also found that there were no reports about preserving non-enzymatic glucose sensors at room temperature for five and a half years. In this paper, we demonstrated that our α-NiS nanosphere films were preserved at room temperature in our laboratory for five and a half years and were still useful for detecting glucose at different concentrations in different solutions (0.1 M NaOH and Krebs buffer).

## Methods

### Preparation of the α-NiS Films

For the α-NiS film fabrication, the synthesis condition was a two-step process: the first step was the fabrication of the nickel nanosheet film [13, 14], and the second step was the synthesis process of the α-NiS film by a physical vapor transport (PVT) method for sulfurizing the nickel nanosheet film [15, 16]. In the first step, nickel nanosheet film was synthesized via a simple electrodeposition method. We used a Pt plane anode and an indium tin oxide (ITO) glass cathode, treated in a cathodic electrodeposition process, for fabricating the nickel nanosheet film. Nickel films were electrodeposited on ITO-coated conducting glass substrates, which were cut into a 0.5 × 1 cm^2^ size. Each one was with a resistance of < 15 Ω/cm^2^. 0.1 M nickel sulfate hexahydrate (NiSO_4_.6H_2_O, Sigma-Aldrich, ≥ 98.5%) and 0.05 M sodium hydroxide (NaOH, SHOWA, 96%) were used to prepare a precursor solution in double-distilled water. We used the deposit nickel film in potentiostatic mode. We set the electrodeposition potential at 3.0 V DC with a solution of pH 7.7. High-quality nickel films were electrodeposited at 40 °C for 10 min. After acquiring nickel films, the nickel nanosheet films were annealed in vacuum-sealed glass ampoules with sulfur sheets. The α-NiS films were annealed at different annealing temperatures (300, 400, and 500 °C) for 4 h. We want to confirm the optimum duration of annealing time, and we annealed the α-NiS films at annealing temperature of 400 °C for different times (3 and 6 h).

### Characterization of the α-NiS Film

The morphology of α-NiS films was characterized by using XRD (SHIMADZU XRD-6000) utilizing Cu Kα radiation, variable vacuum scanning electron microscopy (VVSEM) (HITACHI S-3000N), and FE-SEM/EDS (HITACHI S-4800) at 3.0 kV. The electrochemical properties of α-NiS films were measured by using CV measurements and amperometry with an Ag/AgCl reference electrode by a potentiostat (Jiehan, ECW-5000) in a three-electrode configuration. The α-NiS film was assessed by CV measurements and amperometry in a 15-mL solution of 0.1 M NaOH with different concentrations of glucose. The impedance measurements of α-NiS films were estimated by using an electrochemical impedance spectroscopy (EIS) (Zennium IM6) in 0.1 M KCl containing 1.5 mM Fe(CN)_6_^3−/4−^. The α-NiS film was assessed by CV measurements and amperometry in Krebs buffer (115 mM NaCl, 2 mM KCl, 25 mM NaHCO_3_, 1 mM MgCl_2_, 2 mM CaCl_2_, 0.25% bovine serum albumin [pH 7.4]; equilibrated with 5% CO_2_) [17]. The absorption spectra of the α-NiS films were measured by an UV/Visible/NIR spectrophotometer (HITACHI U-3501) after the α-NiS films were dispersed in distilled water by using a supersonic disperser. The photoluminescence (PL) spectra were obtained by a fluorescence spectrometer (RF-5301PC) with a xenon laser at room temperature. Finally, the crystal structure of the α-NiS nanospheres was investigated by using a HR-TEM (JEOL TEM-2010 HR-TEM) system.

## Results and Discussion

**Fig. 1:**
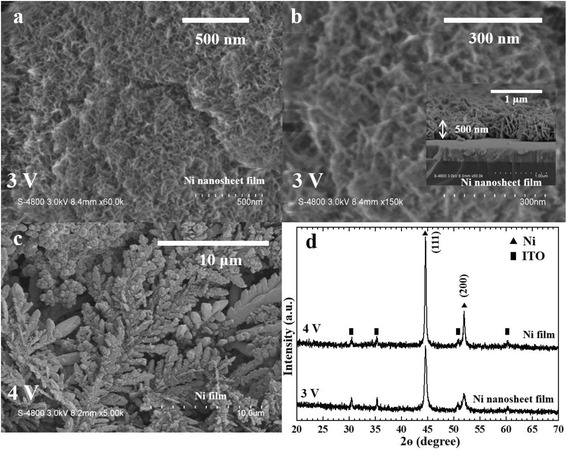
FE-SEM images of the nickel films. **a**, **b** Top view of the nickel nanosheet film was electrodeposited at 3.0 V DC. Inset: cross-section of the nickel nanosheet film. **c** Top view of the nickel film was electrodeposited at 4.0 V DC. **d** The XRD patterns of nickel films were electrodeposited at various potentials (3.0 and 4.0 V DC)

We obtained the nickel nanosheet films by electrodeposition method. We set the DC electrodeposition at the potential of 3.0 V DC and 4.0 V DC. We maintained the electroplating solution at 40 °C for 10 min and observed the electrodepositing nickel film on the ITO glass substrate. Figure 1 showed the results of electrodepositing nickel films. As seen in Fig. 1a, b, the observed surface of the nickel nanosheet film was with an average grain size of 0.01–0.3 μm at the deposition potential of 3.0 V DC. The cross-section of the nickel nanosheet film with the thickness of approximately 500 nm was shown in the inset of Fig. 1b. It was observed that on the surface of the nickel film, it was with an average grain size of 0.5–1.0 μm at the deposition potential of 4.0 V DC. Figure 1d showed the XRD patterns for the nickel films. Diffraction peaks corresponding to XRD patterns for different nickel films were confirmed by comparison with Joint of Committee on Powder Diffraction Standards (JCPDS870712) card. Therefore, we confirmed that the end products were nickel films when the films were observed on the ITO glass substrate.

**Fig. 2:**
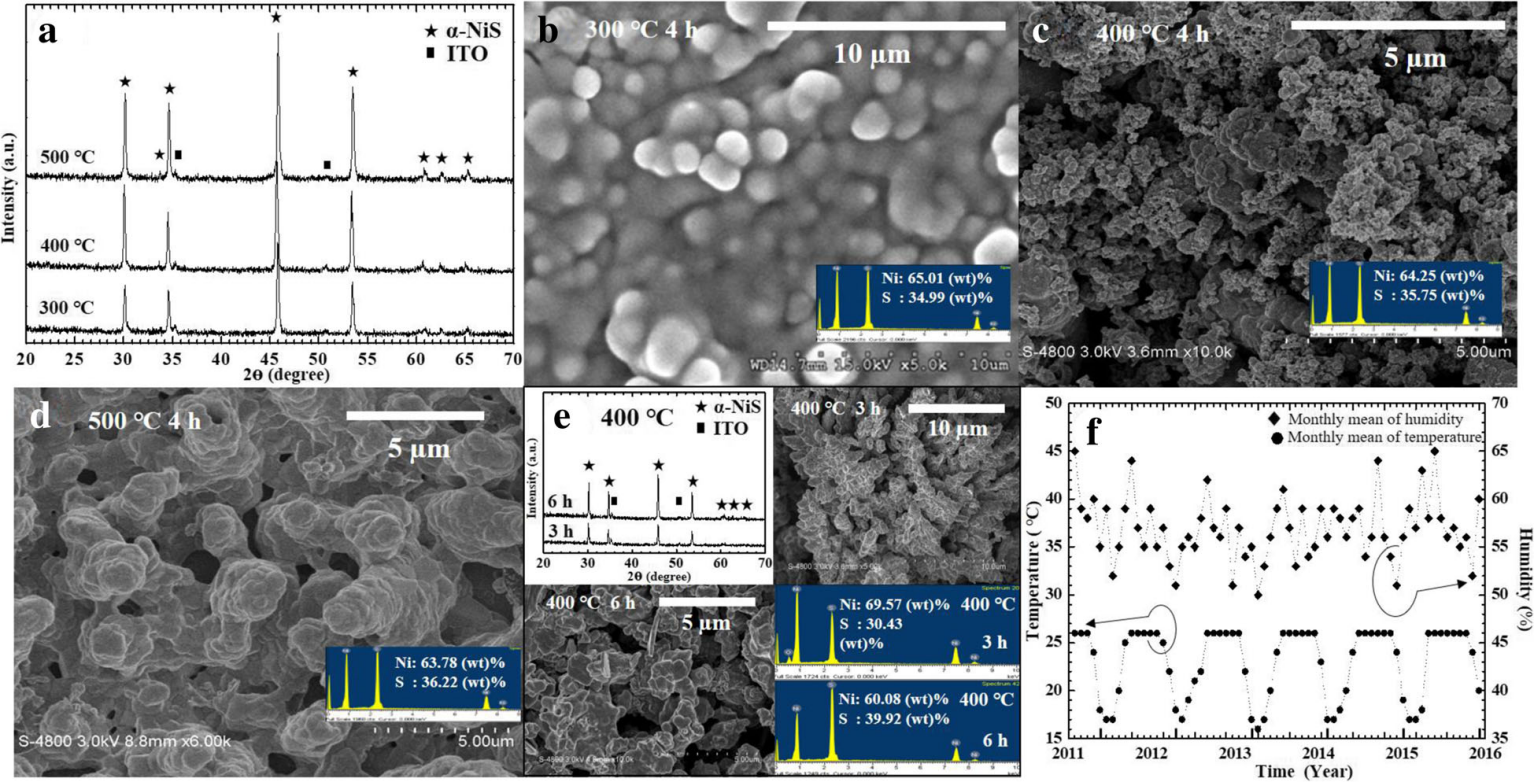
**a** XRD pattern shows the α-NiS nanosphere films at different annealing temperatures (300, 400, and 500 °C). The top view images of the α-NiS films were annealed at **b** 300, **c** 400, and **d** 500 °C for 4 h. Inset: the EDS spectra were in the inset of **b**–**d**. **e** The images showed that XRD patterns (top left), FE-SEM images (top right, 3 h; bottom left, 6 h), and EDS spectra (bottom right) of the α-NiS films at different annealing times (3 and 6 h). **f** The curves showed the record about temperature and humidity measurements in our laboratory for preservation testing of conditions

We considered that the nickel nanosheet film was better than the nickel film for developing the nanostructure of α-NiS film. We sulfurized the nickel nanosheet films in our experiments for getting nano-NiS films. After nickel films were annealed in vacuum-sealed glass ampoules, we got the α-NiS films. Figure 2 showed the results of controlling the different sulfurization temperatures to synthesize α-NiS films. Figure 2a XRD patterns showed that three α-NiS films were synthesized at three different annealing temperatures (300, 400, and 500 °C). In the XRD pattern of each specimen, we observed that diffraction peaks from the different α-NiS films were at the same phase. Diffraction peaks corresponding to XRD patterns of α-NiS films were confirmed by comparison with Joint of Committee on Powder Diffraction Standards (JCPDS750613) cards. Therefore, we confirmed that the end products were α-NiS films. Figure 2b–d showed the different morphologies of the α-NiS films at three different annealing temperatures (300, 400, and 500 °C) for 4 h. The EDS results of α-NiS films with the percentages by weight (wt%) of sulfur (S) and nickel (Ni) elements were shown in the insets of Fig. 2b–d. Figure 2b showed irregularly shaped particles on the surface of the α-NiS film at the annealing temperature 300 °C. We observed the particles to be approximately 0.5–2 μm in Fig. 2b. The EDS result of the α-NiS film at the annealing temperature 300 °C, 34.99 wt% of S, and 65.01 wt% of Ni with a molar ratio of 0.99 (S/Ni) was shown in the inset of Fig. 2b. We observed sphere-like particles and porous structure of α-NiS with an approximate average size of 0.1–0.2 μm on the surface of the α-NiS film at the annealing temperature 400 °C in Fig. 2c. The EDS result of the α-NiS film at the annealing temperature 400 °C, 35.75 wt% of S, and 64.25 wt% of Ni with a molar ratio of 1.02 (S/Ni) was shown in the inset of Fig. 2c. We also observed chain-like particles of α-NiS with an approximate average size of 1–5 μm on the surface of the α-NiS film at the sulfurization temperature 500 °C in Fig. 2d. The EDS result of the α-NiS film at the annealing temperature 500 °C, 36.22 wt% of S, and 63.22 wt% of Ni with a molar ratio of 1.04 (S/Ni) was shown in the inset of Fig. 2c. We observed that the morphologies (irregularly shaped particles, nanospheres, and chain-like particles) of the specimen surfaces changed at different annealing temperatures (300, 400, and 500 °C). In general, we observed different growth evolution and nanostructure formation at the different annealing temperatures. Researchers (Denholme et al.) also presented that the temperature influences the growth kinetics of the NiS_2_ films controlled the varying morphologies by temperature parameter in the Ni-S system [15]. This was due to S vapor pressure. Similarly, it was rationale that the S vapor participated in reactions via vapor-solid or vapor-liquid-solid mechanisms at the Ni metal surface in S vapor and Ni transport reactions. Thus, the reaction was conducted within a closed system and was reliant on the vapor pressure of the reactants. The vapor pressure was dependent upon the reaction temperature and the stoichiometric ratio of the reactants. We thought that the varying morphologies of NiS significantly in S vapor pressure increased as temperatures increased with different enhancements of Ni and S reaction rate.

We also want to confirm the optimum duration of annealing time. The α-NiS films were annealed at 400 °C for other times (3 and 6 h). The results were shown in Fig. 2e. We observed that the XRD patterns of the different α-NiS films were at the same phase and were confirmed by JCPDS750613 cards in the inset (top left) of Fig. 2e. We observed the particles to be approximately 0.5–1 μm on the surface of the α-NiS film at the sulfurization temperature 400 °C for 3 h in the inset (top right) of Fig. 2e. The EDS result of the α-NiS film at the annealing temperature 400 °C, 30.43 wt.% of S, and 69.57 wt.% of Ni for 3 h with a molar ratio of 0.8 (S/Ni) was shown in the inset (bottom right) of Fig. 2e. We observed the particles to be approximately 0.5–2 μm on the surface of the α-NiS film at the sulfurization temperature 400 °C for 6 h in the inset (bottom left) of Fig. 2e. The EDS result of the α-NiS film at the annealing temperature 400 °C, 39.92 wt.% of S, and 60.08 wt.% of Ni for 6 h with a molar ratio of 1.21 (S/Ni) was shown in the inset (bottom right) of Fig. 2e. As seen in the inset (EDS result) of Fig. 2c, it showed that there was no excess or lack of S for the 4-h specimen, which was close to the stoichiometric ratio of 1 (S/Ni). Finally, the SEM image of Fig. 2c having more nanospheres on the surface of α-NiS film for the annealing time 4 h was compared with two SEM images for different annealing times (3 and 6 h) with larger particles in the insets (top right and bottom left) of Fig. 2e. We confirmed that the optimum duration of annealing time was 4 h.

**Fig. 3:**
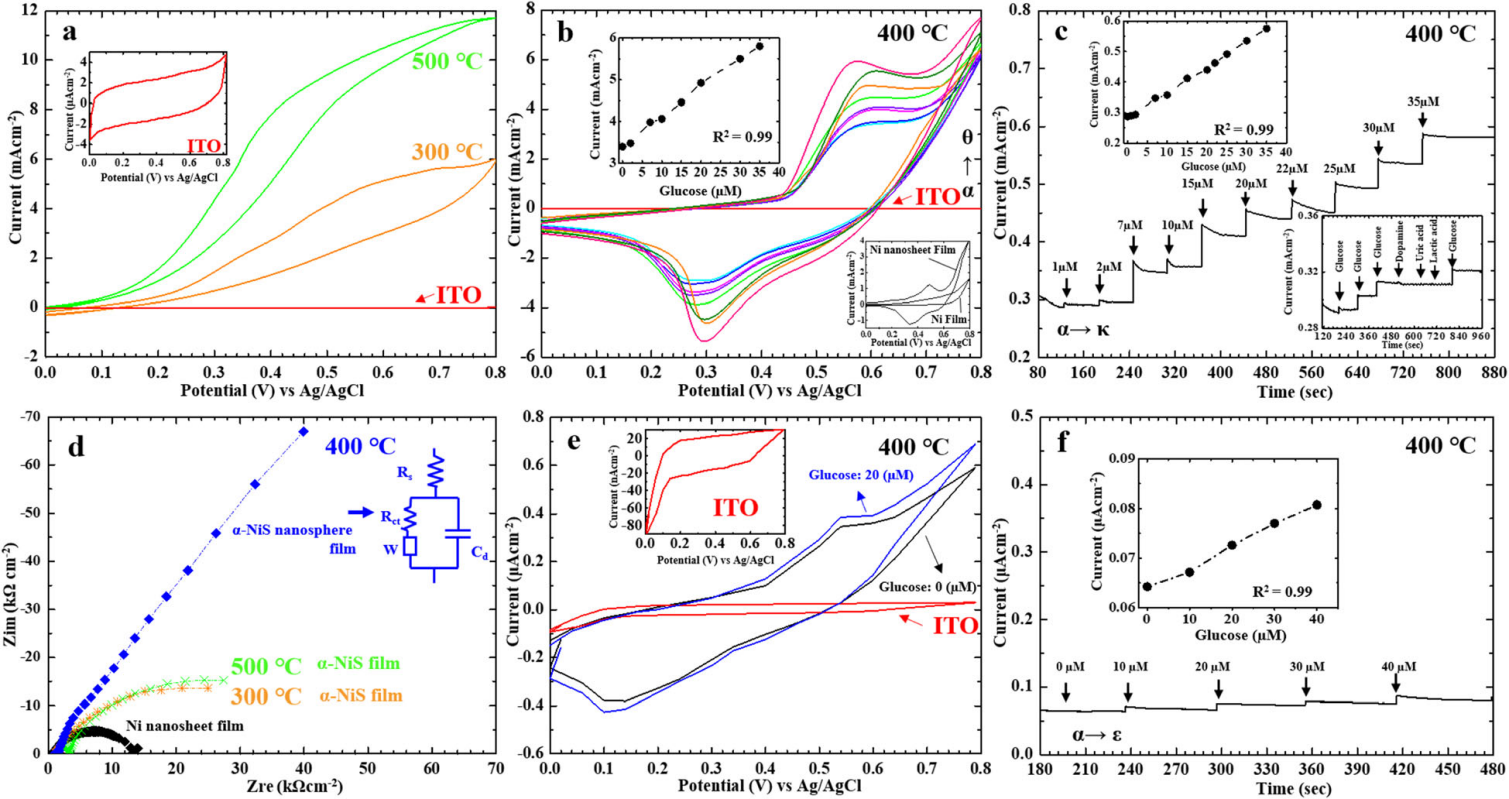
**a** Three CVs in the image: the red curve showed the CV of bare ITO; the orange and green curves were the CVs of α-NiS films at different annealing temperatures (300 and 500 °C). Inset: CV of bare ITO/glass. **b** CV of nano-NiS/ITO in 0.1 M NaOH with different concentrations of glucose: (α) 0 μM, (β) 2 μM, (γ) 7 μM, (δ) 10 μM, (ε) 15 μM, (ζ) 20 μM, (η) 30 μM, and (θ) 35 μM. Inset: top left—plot of oxidation peak current against glucose concentration; bottom—CVs of Ni film and Ni nanosheet film. **c** The α-NiS nanosphere film was assessed by amperometry in 0.1 M NaOH with different concentrations of glucose: (α) 1 μM, (β) 2 μM, (γ) 7 μM, (δ) 10 μM, (ε) 15 μM, (ζ) 20 μM, (η) 22 μM, (θ) 25 μM, (ι) 30 μM, and (κ) 35 μM. Inset: top left—plot of the current responses against glucose concentrations; bottom—chronoamperometric response of NiS/ITO in 0.1 M NaOH with 2 μM glucose and in the presence of 2 μM dopamine, uric acid, and lactic acid at an applied potential of 0.6 V DC. **d** Nyquist plots of the nickel nanosheet film, α-NiS nanosphere film, and α-NiS films at different annealing temperature (300 and 500 °C) in 0.1 M KCl containing 1.5 mM Fe(CN)_6_^3−/4−^. **e** CV of nano-NiS/ITO in Krebs with different concentrations of glucose: (α) 0 μM and (β) 20 μM. Inset: top left—CV of bare ITO/glass. **f** The α-NiS nanosphere film was assessed by amperometry in Krebs buffer with different concentrations of glucose: (α) 0 μM, (β) 10 μM, (γ) 20 μM, (δ) 30 μM, and (ε) 40 μM. Inset: top—plot of the current responses against glucose concentrations

After synthesizing α-NiS nanosphere films, we placed some of the α-NiS nanosphere films in small plastic containers with plastic covers in our laboratory with the air condition for five and a half years. The time of the preservation test for our α-NiS nanosphere films was from 1 August 2011 to 31 December 2016. As seen in Fig. 2f, the curves showed the temperature (16–26 °C) and relative humidity (50–65%) which were recorded in our laboratory for preservation test from 1 August 2011 to 31 December 2016. After finishing the preservation test, we wanted to confirm the α-NiS nanosphere films which still had the current responses at different glucose concentrations by CV measurements and amperometry in a solution in January 2017. We surveyed some papers about measuring the electrochemical behavior of NiS specimen for a non-enzymatic glucose sensor. Many researchers measured the specimens by CV measurements and amperometry in a 0.1 M NaOH solution because they compared the results with the same condition easily [8–12]. Figure 3 showed the CV and amperometry properties of α-NiS films. Regarding area of working electrode was 0.2 × 0.5 cm^2^ for detecting glucose on the surface of α-NiS nanosphere film in all experiments. The oxidation-reduction (redox) reaction of the α-NiS films was estimated by using the CV method by an Ag/AgCl reference electrode with a potentiostat. The CV characteristics of α-NiS films were scanned between 0 and 0.8 V for 1 cycle by a potentiostat. The specimens were measured in a three-electrode configuration at the scan rate of 20 mVs^−1^. Regarding the concentration of NaOH, we chose 0.1 M for the solution because we saw the following formula (1) that the more OH^−^ anions we had, the more e^−^ anions in solution [8].

1 $$ \mathrm{NiS}+{\mathrm{OH}}^{-}\leftrightarrow \mathrm{NiS}\mathrm{OH}+{\mathrm{e}}^{-} $$

According to the above formula (1), we considered that the more e^−^ anions we had in a solution, the larger current value showed in a potentiostat. There were three curves in Fig. 3a. The red CV curve of the bare ITO was shown in the inset of Fig. 3a. The orange and green CV curves were redox reaction of the α-NiS films at different annealing temperatures (300 and 500 °C). We observed that the CV curves did not have negative reduction potentials in Fig. 3a. We also found that two α-NiS films did not have any current responses to different glucose concentrations. As seen in Fig. 3b, it showed that the α-NiS nanosphere film was assessed by CV measurements in a solution of 0.1 M NaOH with different glucose concentrations (2, 7, 10, 15, 20, 30, and 35 μM) at a scan rate of 20 mVs^−1^. Obviously, we saw the redox potential of the α-NiS nanosphere film in Fig. 3b. The similar redox curves of nano-NiS film were found in the other paper [8]. Researchers (Padmanathan et al. 2015) reported that the explanation of reaction mechanism was the two redox Eqs. (2) and (3) about sensing glucose of nano-NiS film. The two equations were shown below [8]:

2 $$ {\mathrm{Ni}}^{\mathrm{II}}\to {\mathrm{Ni}}^{\mathrm{II}\mathrm{I}}+{\mathrm{e}}^{-} $$

3 $$ {\mathrm{Ni}}^{\mathrm{II}\mathrm{I}}+\mathrm{glucose}\to {\mathrm{Ni}}^{\mathrm{II}}+\mathrm{gluconolactone} $$

As seen in Fig. 3b, the different current values of oxidation peaks were changed at 0.6 V obviously. We observed that a dotted line had a linear relationship about the different current responses of oxidation peaks against different glucose concentrations in the inset (left) of Fig. 3b. The CV curves for the nickel nanosheet film and nickel film were also shown in the inset (bottom) of Fig 3b. The current responses of CV curve for the nickel nanosheet film were larger than Ni film from 0 to 0.8 V in the inset (bottom) of Fig. 3b. We considered that we used the nickel nanosheet film for a precursor in the synthesizing process of α-NiS nanosphere film, and we had more opportunities to get larger current responses in the CV curve. Figure 3c showed that the different current responses of α-NiS nanosphere film were for detecting glucose at different concentrations (1, 2, 7, 10, 15, 20, 22, 25, 30, and 35 μM) by amperometry. We observed the different current responses of the glucose concentrations from 1 to 35 μM with a linear relationship having a correlation coefficient of 0.99 in the inset (left) of Fig. 3c. It was described by:

4 $$ I\left[{\mathrm{mAcm}}^{-2}\left]=0.0084\right[\mathrm{glucose}\right]\upmu \mathrm{M}+0.2821 $$

The sensitivity value was estimated at 8.4 μA μM^−1^ cm^−2^ for the Eq. (4). The chronoamperometric response of α-NiS nanosphere film in 0.1 M NaOH with 2 μM glucose and 2 μM dopamine, 2 μM uric acid, and 2 μM lactic acid at an applied potential of 0.6 V DC were shown in the inset (bottom) of Fig. 3c. We demonstrated that our α-NiS nanosphere film was a non-enzymatic glucose sensor in 0.1 M NaOH with anti-interference ability towards dopamine, uric acid, and lactic acid.

Regarding the electrochemical results on the α-NiS nanosphere films, we considered that only 400 °C specimen showed many small nanoparticles and porous structure on the surface of α-NiS nanosphere film in Fig. 2c. The smaller nanoparticles and porous structure were deposited on the surface of the α-NiS nanosphere film, so the nanosphere film provided a larger surface area and higher responses in electrochemical detection. We observed that the specimens were annealed at 400 °C for 4 h with the current responses at low glucose concentrations. Only 400 °C specimen having the good glucose response was due to many small nanoparticles and porous structure on the surface of α-NiS nanosphere film.

Figure 3d showed that the electrochemical impedance spectroscopy (EIS) of α-NiS films was detecting in a solution of 0.1 M KCl (containing 1.5 mM Fe(CN)_6_^3−/4−^). We observed that the Warburg (*W*) impedance of α-NiS nanosphere film was larger than two other α-NiS films. The elements of EIS model of α-NiS nanosphere film were *R*_s_ = 133 Ω, *R*_ct_ = 42.1 Ω, *C*_d_ = 22.1 μF, and *W* = 11.7 kΩ. The electrochemical impedance of Ni nanosheet film was also shown in Fig. 3d, and it had the lower impedance value in these patterns. We also calculated the values of our non-enzymatic glucose sensor for stability, standard deviation (SD) of stability, and reusability (see Table 1). From the values of the SD of stability in Table 1, we observed that the average stability value (0.011 mA/min) of measurement 14 times was larger than the average stability value (0.006 mA/min) of measurement 13 times. We believed that numerical value of reusability was approximately 13 (SD ≤ 0.002 mA/min).

**Table 1:** Calculation of the values of the α-NiS nanosphere films for detecting 20 μM glucose for average stability, standard deviation (SD), and reusability

Time of test	Average of the initial current at 1 s (mA)	Stability of specimen 1 at 1 min (mA/min)	Stability of specimen 2 at 1 min (mA/min)	Average value of stability 13 and 14 times (mA/min)	Standard deviation (SD) value 13 and 14 times (mA/min)	Reusability (SD ≤ 0.002)
1st	0.442	0.003	0.004			
2nd	0.444	0.003	0.004			
3rd	0.440	0.004	0.005			
4th	0.443	0.004	0.006			
5th	0.441	0.005	0.006			
6th	0.439	0.004	0.007			
7th	0.438	0.005	0.007			
8th	0.441	0.005	0.008	0.006/0.011	0.002/0.019	13
9th	0.439	0.005	0.008			
10th	0.438	0.005	0.009			
11th	0.437	0.006	0.009			
12th	0.436	0.008	0.010			
13th	0.434	0.009	0.011			
14th	0.391	0.069	0.084			
15th	0.308	0.109	0.128			

*N* = 2

After finishing the measurement for the electrochemical behavior of NiS specimen in 0.1 M NaOH, we also surveyed many papers for a physiological condition. Those researchers used different solutions such as phosphate-buffered saline (PBS), annexin V binding buffer, aECF solution, and Krebs buffer for application of cell culture [17–21]. Some researchers selected Krebs buffer for cell culture buffer at low glucose concentration [20, 21]. The linear range of our α-NiS nanosphere film for detecting low glucose consecration was from 1 to 35 μM in 0.1 M NaOH, so it had a practical significance for us that using our sensor for detecting low glucose consecration in Krebs buffer for a physiological condition. The α-NiS nanosphere film was used to detect glucose at different concentrations in Krebs buffer. We used our α-NiS nanosphere film to detect at the different glucose concentrations (0 and 20 μM) by cyclic voltammogram (CV) in Krebs buffer (115 mM NaCl, 2 mM KCl, 25 mM NaHCO_3_, 1 mM MgCl_2_, 2 mM CaCl_2_, 0.25% bovine serum albumin [pH 7.4]; equilibrated with 5% CO_2_, adjusted to pH 7.4 with 0.01 M NaOH) [20]. As seen in the inset of Fig. 3e, it showed the background CV curve of bare ITO. Figure 3e also showed the CV curves of NiS/ITO electrode in Krebs buffer containing 0 and 20 μM of glucose. We observed the CV curves with different current responses near 0.6 V obviously. As seen in Fig. 3f, the α-NiS nanosphere film was assessed by amperometry in Krebs buffer (adjusted to pH 7.4 with 0.01 M NaOH) for detecting different glucose concentrations: (α) 0 μM, (β) 10 μM, (γ) 20 μM, (δ) 30 μM, and (ε) 40 μM. The inset figure showed the plot of oxidation peak current against glucose concentration. A curve of the amperometric response was shown in the inset (top) of Fig. 3f which was demonstrating a linear relationship with a correlation coefficient of 0.99. It was described by I[μAcm^−2^] = 0.0004[glucose]μM + 0.0638.

**Fig. 4:**
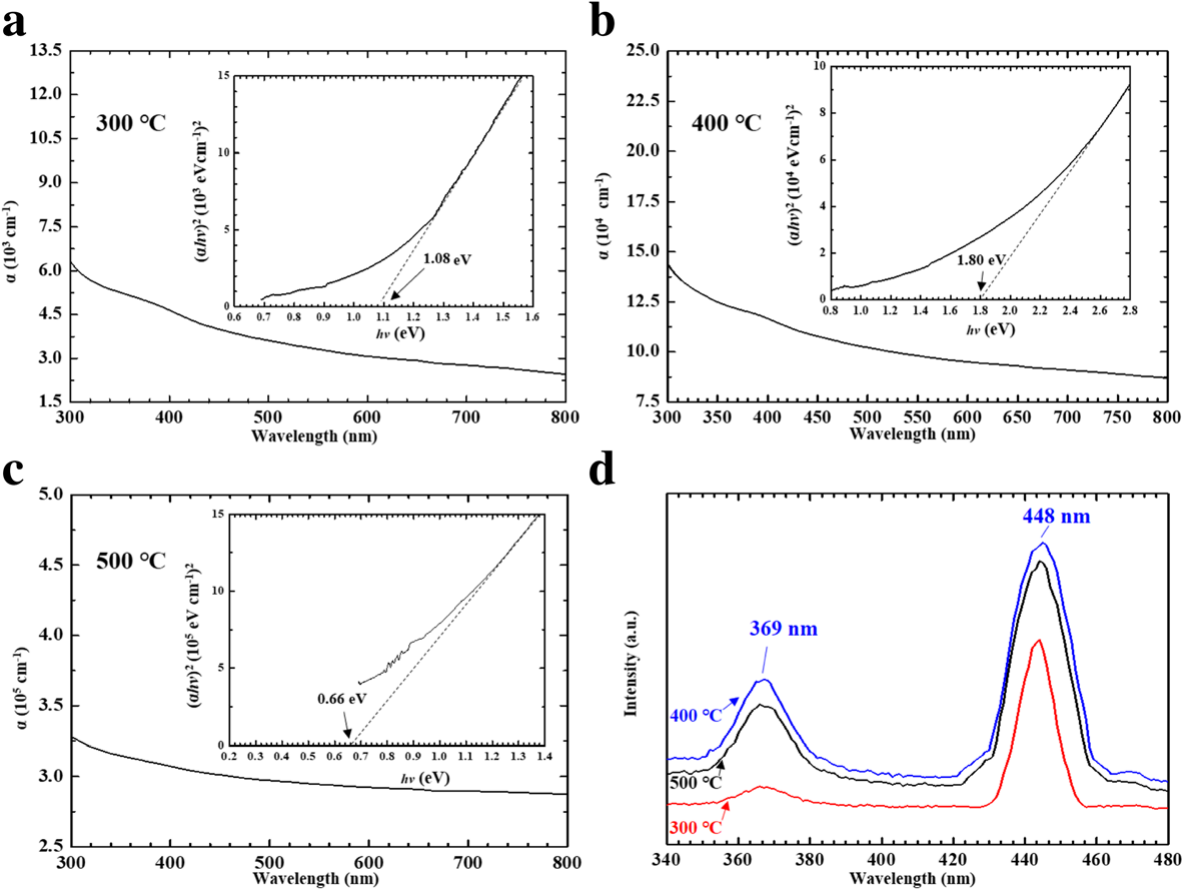
UV/Visible/NIR absorption spectra and (*αhν*)^2^ versus *hν* plot in the insets of figures for synthesizing α-NiS films at **a** 300, **b** 400, and **c** 500 °C. **d** Fluorescence spectra of the α-NiS films were fabricated at different annealing temperatures (300, 400, and 500 °C for 4 h)

Figure 4 showed the UV/Visible/NIR absorption and fluorescence spectra. We recorded the UV/Visible/NIR absorption of the α-NiS films in the spectral range of 300–800 nm (Fig. 4a–c) for different annealing temperatures (300, 400, and 500 °C). To determine the energy gap (*E*_g_) of the nanospheres, the following dependence of absorption coefficient (*α*) on the photon energy equation was used [22]:

5 $$ \alpha hv=A{\left( hv-{E}_{\mathrm{g}}\right)}^m $$

where *E*_*g*_ was the energy gap, *A* was the constant having separate values for different transitions, *hν* was the photon energy, and *m* was an exponent that assumed the values 1/2, 3/2, 2, and 3 which were interrelated to the nature of the electronic transition. It was responsible for the absorbance. It showed the (*αhν*)^2^ against *hν* plot in the inset of Fig. 4a–c. When *m* = 1/2, these absorption spectra of α-NiS films allowed the proper values for direct transition. As seen in the inset of Fig. 4a–c, we estimated three energy gap (*E*_g_) values (1.08, 1.8, and 0.66 eV) of the α-NiS films. We used dotted lines to fit the curves from 0.6 to 2.8 eV in the inset of Fig. 4a–c. As seen in the inset of Fig. 4a–c, we also observed that the highest energy gap (*E*_g_) of α-NiS nanosphere film was approximately 1.8 eV at the annealing temperature 400 °C. This study also used fluorescence equipment to investigate the optical properties of the specimens. Previous researchers focused on the fluorescence spectra of the α-NiS particles which were influenced by the different phases, shapes, structures, and the surface/volume ratio [23]. As seen in Fig. 4d, we observed the fluorescence spectra of α-NiS films having ultraviolet emissions at different annealing temperatures (300, 400, and 500 °C). PL spectra of the specimens showed the sharp emission peaks at 448 nm and the emission peaks at 369 nm (excited at *λ*_ex_ = 277 nm) [23, 24]. According to the results on the optical properties of our α-NiS films, we considered that different annealing temperatures had a chance to get different grain size on the NiS film. Regarding the nanoparticles exhibiting quantum confinement, increasing the nanoparticle of size influenced the bandgap decreasing with the temperature from 400 to 500 °C [25]. The optical properties of NiS changed with different grain size, so the optical properties of NiS significantly changed with different temperatures [25]. The varying optical properties of NiS film significantly with different temperatures should be due to exhibiting size effect, decreasing the particle size influenced on the bandgap.

**Fig. 5:**
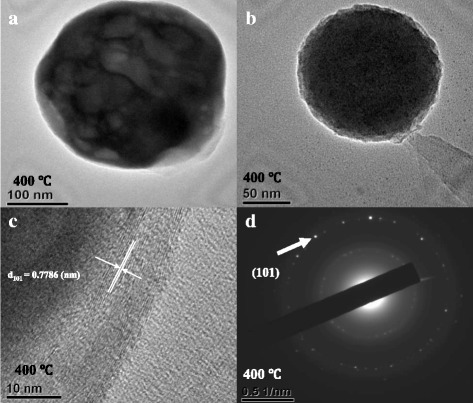
**a**–**c** HR-TEM images of the α-NiS nanosphere. **d** SAED pattern of the α-NiS nanosphere was annealing at 400 °C for 4 h

We considered focusing HR-TEM analysis on α-NiS nanosphere film because we got many α-NiS nanospheres for the non-enzymatic glucose sensors at the annealing temperature 400 °C. As seen in Fig. 5, we observed that the α-NiS nanospheres were annealed at 400 °C for 4 h. The information on the microstructure of as-prepared α-NiS nanosphere was obtained by HR-TEM. Figure 5a, b revealed HR-TEM images of the nanospheres. The diameter of the nanosphere was from 150 to 250 nm. Figure 5c HR-TEM image also showed clear lattice fringes with an interspace of 0.7786 nm which were corresponding to the distance between two adjacent (101) planes of the α-NiS nanosphere. Figure 5d showed a SAED pattern of the nanosphere, and the spots of the diffraction ring was indexed to (101) of the α-NiS nanostructure.

## Conclusion

In summary, the α-NiS nanosphere films were investigated by using XRD, VVSEM, FE-SEM, EDS, EIS, UV, PL, and HR-TEM equipment. We observed that the α-NiS nanosphere film was formed by controlling the annealing temperature at 400 °C for 4 h in vacuum-sealed glass ampoules. The energy gap (*E*_g_) of the α-NiS nanosphere film was approximately 1.8 eV. After preserving our α-NiS nanosphere films in our laboratory for five and a half years, we observed that the α-NiS nanosphere films still had the current responses at different glucose concentrations by CV measurements and amperometry in different solutions (0.1 M NaOH and Krebs buffer). The linear range of detecting glucose was from 1 to 35 μM in 0.1 M NaOH. For a physiological condition, the linear range of detecting glucose was approximately from 0 to 40 μM in Krebs buffer.

## Abbreviations

CV: Cyclic voltammogram
EDS: Energy-dispersive spectrometer
FE-SEM: Field emission scanning electron microscopy
HR-TEM: High-resolution transmission electron microscopy
NiS: Nickel sulfide
PL: Photoluminescence
PVT: Physical vapor transport
SD: Standard deviation
UV/Visible/NIR: Ultraviolet/visible/near-infrared
VVSEM: Variable vacuum scanning electron microscopy
wt%: Percentage by weight
XRD: X-ray diffraction
